# A single-dose of oral nattokinase potentiates thrombolysis and anti-coagulation profiles

**DOI:** 10.1038/srep11601

**Published:** 2015-06-25

**Authors:** Yuko Kurosawa, Shinsuke Nirengi, Toshiyuki Homma, Kazuki Esaki, Mitsuhiro Ohta, Joseph F. Clark, Takafumi Hamaoka

**Affiliations:** 1 Department of Sport and Health Science, Ritsumeikan University, Kusatsu, Shiga, Japan; 2 Department of Economics, Tokuyama University, Shunan, Yamaguchi, Japan; 3 Department of Clinical Chemistry, Kobe Pharmaceutical University, Kobe, Hyogo, Japan; 4 Department of Neurology, University of Cincinnati, Cincinnati, Ohio, USA

## Abstract

Our aim was to determine the quantitative effects of a single-dose of Nattokinase (NK) administration on coagulation/fibrinolysis parameters comprehensively in healthy male subjects. A double-blind, placebo-controlled cross-over NK intervention study was carried out in 12 healthy young males. Following the baseline blood draw, each subject was randomized to receive either a single-dose of 2,000 FU NK (NSK-SD, Japan Bio Science Laboratory Co., Ltd) or placebo with subsequent cross-over of the groups. Subjects donated blood samples at 2, 4, 6 and 8 hours following administration for analysis of coagulation/fibrinolysis parameters. As a result, D-dimer concentrations at 6, and 8 hours, and blood fibrin/fibrinogen degradation products at 4 hours after NK administration elevated significantly (p < 0.05, respectively). Factor VIII activity declined at 4 and 6 hours (p < 0.05, respectively), blood antithrombin concentration was higher at 2 and 4 hours (p < 0.05, respectively), and the activated partial thromboplastin time prolonged significantly at 2 and 4 hours following NK administration (p < 0.05 and p < 0.01, respectively). All the changes, however, were within the normal range. In conclusion, thus, a single-dose of NK administration appears enhancing fibrinolysis and anti-coagulation via several different pathways simultaneously.

Fibrinolytic enzymes have been derived from different microorganisms, including the genus *bacillus* which occurs in traditional fermented foods. Nattokinase (NK) is an enzyme contained in the sticky component of natto, a cheese-like food made of soybeans fermented with *Bacillus subtilis*. Natto has a long cultural history, extending back more than 1,000 years in Japan, and the fibrinolytic mechanism of NK has been explored more extensively than of other microbial fibrinolytic enzyme^1^. NK is a serine protease that has 275 amino acid residue with a molecular weight of approximately 28 kDa^2^ and exhibits a high homology with *subtilisins* E (99.5%)^3^ and *subtilisins* J (Amylosacchariticus, 99.3%)^4^. Using a clot lysis assay *in vitro*, the cleavage of cross-linked fibrin by NK was 6 times more efficient than by plasmin as measured from kcat/Km^5^. At an equivalent molar ratio of doses (0.12 μmol/kg), NK is four times more effective than plasmin at dissolving a thrombus in rats *in vivo*^6^. These facts suggest that NK has strong fibrinolytic activity both *in vitro* and *in vivo*. In *in vitro* studies, NK not only directly cleaves cross-linked fibrin, but also activates the production of tissue-type plasminogen activator (tPA), resulting in the transformation of inactive plasminogen to active plasmin^5^. NK also enhances its fibrinolysis through cleavage and inactivation of plasminogen activator inhibitor-1 (PAI-1) *in vitro*, which is the primary inhibitor of tPA and regulates total fibrinolytic activity by its relative ratio with tPA^7^. Several animal studies also demonstrated the efficacy of NK on thrombolysis *in vivo*. Restoration of blood flow was directly proportional to the amount of NK injected into chemically induced thrombi in rats^6^. Another study demonstrated that blood circulation was recanalized completely after 5 hours of oral NK administration in dogs^8^. Although NK has been promoted as a most promising microorganism-derived enzyme for reducing thrombosis risk, the mechanism by which NK accelerates fibrinolysis has not been fully determined, and the data in humans is still limited. The purpose of this double-blind, placebo-controlled cross-over study was to comprehensively determine the effects of a single-dose of NK administration on coagulation/fibrinolysis profile in healthy young Japanese.

## Materials and Methods

The study was approved by the Institutional Review Board for Human Experiments, Ritsumeikan University, in accordance with the ethical principles contained in the Declaration of Helsinki. Written informed consent was obtained from all twelve participants. All subjects were healthy, young male Japanese (age; 22.3 ± 0.6 years old, Height; 169.3 ± 1.0 cm, body weight; 61.9 ± 1.5 kg, BMI; 21.6 ± 0.5, %body fat; 12.7 ± 0.9%) with no medication. None of the participants had a history of hematologic disease, symptoms of venous or arterial diseases. All subjects stated they had never taken NK supplement before, and were not consuming food natto within 2 months before the experiments started. The same researchers performed all the procedures using identical techniques.

### Experimental Procedures

**Figure 1:**
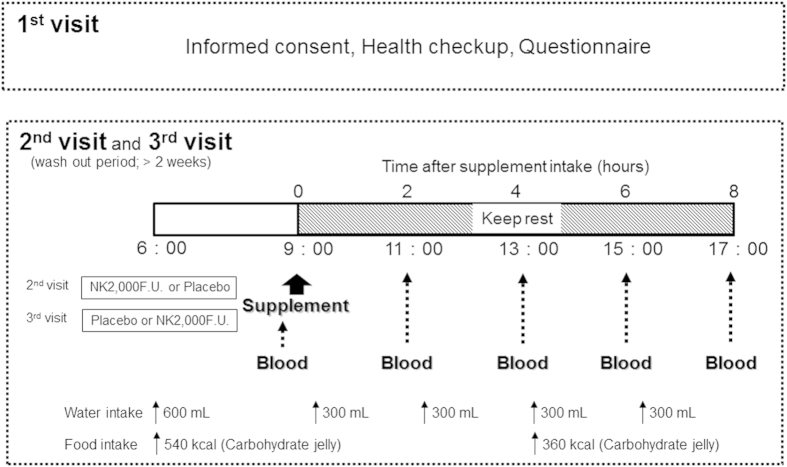
This figure shows the study design and the experimental procedures.

A double-blind, placebo-controlled cross-over NK intervention study was carried out in twelve healthy subjects. Baseline blood samples were collected between the hours of 8:30 AM and 9:30 AM to minimize potential diurnal variations. Following the baseline blood draw, each subject was randomized to receive either a single-dose of 2,000 FU NK in a soft gel capsule form (NSK-SD, Japan Bio Science Laboratory Co., Ltd, Osaka, Japan) or soft gel capsule containing the placebo (P). Subjects donated blood samples at 2, 4, 6 and 8 hours following administration for coagulant/fibrinolysis parameters analysis. After a washout period more than 2 weeks, the second trial commenced with the alternate group assignment. During the experiment, the time and quantity of water and caloric intake were identical in both groups (Fig. 1). No side effects were declared.

### Blood sampling and analysis

Blood samples from the median cubital vein were drawn following smooth venipuncture employing minimal stasis, and stored in siliconized glass tubes with 3.2% trisodium citrate (VenoJect; Terumo, Tokyo, Japan), EDTA (VenoJect), sodium fluoride (VenoJect) or without anticoagulant agent as appropriate. Whole blood was used for platelet count/blood cell count/leukocyte analysis. Plasma for the thrombophilia/fibrinolytic investigations were obtained after centrifugation at 2800 *g* at 4 °C for 20 minutes and stored at minus 80 °C until analysis of fibrin/fibrinogen degradation products (FDP, latex photometric immunoassay, LPIA), D-dimer (LPIA), total plasminogen activator inhibitor-1 (Total PAI-1, latex agglutination-turbidimetric immunoassay), plasminogen antigen (latex agglutination-turbidimetric immunoassay), plasminogen activity (synthesized substrate assay), the activated partial thromboplastin time (aPTT, blood coagulation time method), the prothrombin time (PT, Quick’s method), fibrinogen (thrombin method), blood coagulation factor VIII activity (F VIII activity, aPTT method), blood coagulation factor VII activity (F VII activity, PT method), antithrombin (AT, LPIA), and plasmin-α2 plasmin inhibitor complex (PIC, LPIA). White blood cell counts (WBC, flow cytometry), platelet (PLT) and red blood cell counts (RBC, electric resistance measurement), blood glucose (enzymatic method) and serum total protein concentration (Biuret test) were also measured. All assays were done by automated analytical system (Mitsubishi chemical medience, Tokyo, JAPAN) at one time to reduce potential variation among lots.

### Data analysis

The effects of NK administration were analyzed by two-way repeated measures analysis of variance between groups. Blood data taken after the supplementation were compared between groups by a multiple comparison using Bonferroni’s correction. Quantitative results are represented as means ± standard error. Statistical analyses were performed using the Japanese version of SPSS v.20 (IBM SPSS Japan, Tokyo, Japan) and a *p*-value of <0.05 was considered to be statistically significant.

## Results

In this study, we measured each blood parameter until 8 hours after administration, in reference to our preliminary trials which represented all the average peak values in blood appeared within 8 hours after NK intake in 6 young males.

**Figure 2:**
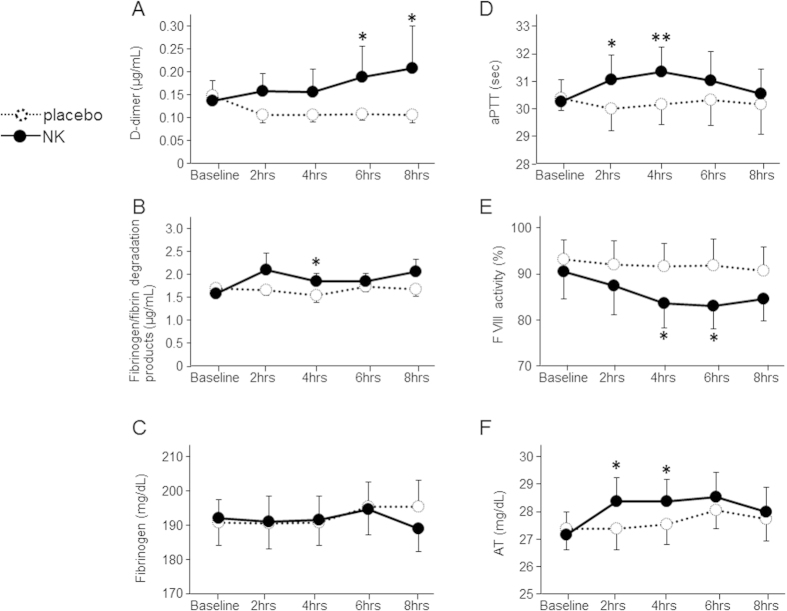
The figures show the fibrinolysis/coagulation parameters before and after a 2,000 FU of NK administration or placebo in twelve healthy young male, double blind crossover placebo-controlled design. Data are expressed as mean ± SEM. Statistically significant when compared with placebo: *P < 0.05, **P < 0.01.

D-dimer, the subunit of the specific degradation products of cross-linked fibrin, and FDP elevated following NK administration (+44.5 ± 12.9% for 6 hours, +38.2 ± 19.2% for 8 hours in D-dimer, +21.2 ± 6.3% for 4 hours in FDP compared with baseline values, p < 0.05 for placebo, respectively, Fig. 2A,B). The aPTT prolonged significantly at 2 and 4 hours following NK supplementation (p < 0.05 and p < 0.01, respectively, Fig. 2D). Factor VIII activity declined at 4 and 6 hours following NK intake (−7.4 ± 1.9% and −7.6 ± 1.6% on average compared with baseline data, p < 0.05 for placebo, respectively, Fig. 2E). Antithrombin concentration increased significantly after NK administration (p < 0.05 for 2 and 4 hours, Fig. 2F). All the blood data changed after NK administration, however, were within the normal range. No differences were observed in any other parameters between NK and placebo groups at any time points before and after supplementation (Fig. 2C, Table 1).

## Discussion

This study provides the first evidence of NK’s ability to enhance fibrinolysis and antithrombosis contemporaneously after a single-dose of oral NK administration in human.

### Bioavailability

NK is effectively absorbed across the rat intestinal tract inducing fibrinolysis after intraduodenal administration^6^. Recently, Ero and colleagues presented the first bioavailability data of NK in human by enzyme-linked immunosorbent assay^9^. Following 2,000 FU NK administration (the same amount as in our current study), they demonstrated NK serum activity between 2 through to 24 hours in healthy subjects. Our data, which confirmed an increase in activity of fibrinolysis and anticoagulant parameters between 2 and 8 hours after NK intake, is consistent with their results.

### Fibrinolysis parameters

NK’s fibrinolytic potential was first identified *in vitro* study using fibrin plate^10^. The NK had 6 times stronger activity *in vitro*^5^, and approximately 4 times greater efficiency for cleavage of cross-linked fibrin as compared with plasmin in rats^6^. Of note, among subtilisins, only NK demonstrates high substrate specificity for fibrin^1^, despite high homology^2^,^5^. In this study, we found that a single-dose of NK administration enhances fibrinolysis via cleavage of cross-linked fibrin, and its effect lasted for a relatively long period of time (over 8 hours), compared with tissue-type plasminogen activator’s (t-PA) and/or urokinase’s 4–20 minutes half-life in human blood.

### Coagulation parameters

The decline of factor VIII activity after NK intake were similar with previous data in which three different subject groups (healthy individuals, patients with cardiovascular risk factors, and patients undergoing dialysis) took 4,000 FU of NK daily for 2 months, and factor VIII antigen declined significantly after 2 month of NK intake in all three groups^11^. The precise mechanism of this NK action is not yet clarified. However, to the extent that elevation of Factor VIII level is known to be risk factors for cardiovascular and related diseases^12^, we propose here further potential for acute effects of NK to reduce the risk of thrombosis.

In this study, we found NK administration increased blood antithrombin concentration after 2 and 4 hours of administration. To the best of our knowledge, this is a novel pathway of NK’s antithrombotic action and there is no data available to reveal the precise mechanism how NK enhances blood antithrombin levels. Only few studies were published which demonstrated soybean fermented with *basidiomycete* facilitated antithrombotic activity indirectly via elongation of prothrombin time, however, the mechanism is still unknown^13^. Esmon, *et al.* emphasize that there is a strong molecular links between inflammation and coagulation, and this crosstalk creates a cycle that progresses to vascular injury as occurred in septic shock^14^. Thrombin, Factor Xa and tissue factor are the main targets for the treatment of this disease. Antithrombin is one of the most important physiological regulators for blood coagulation cascade, with the role of inhibition of thrombin, factors Xa and IXa^15^. In fact, antithrombin activity after treatment of antithrombin administration was the predictor of prognosis in patients with septic disseminated intravascular coagulation^16^. Antithrombin’s anti-inflammatory effects via elevation of cyclic AMP^17^,^18^, therefore, NK administration might improve prognosis in patients who suffer from infectious or other inflammatory diseases.

**Figure 3:**
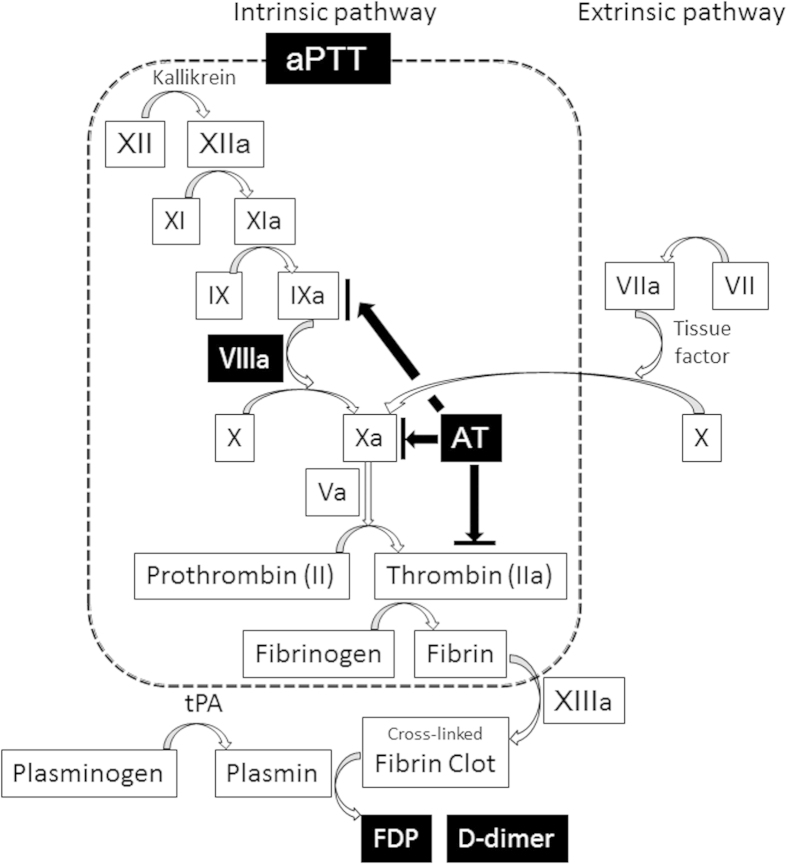
This figure shows the diagram of blood coagulation/fibrinolysis cascade. White colored factors with black background were altered by a single-dose of NK administration.

All drugs currently approved and/or under clinical investigation for treatment of thrombolysis function as plasminogen activators, which lead to proteolytic degradation of fibrin clots mediated by plasmin alone. However, NK activates multiple fibrinolytic and anti-thrombotic pathways simultaneously, either directly or indirectly (Fig. 3). In general, a thrombolytic agent requires small amount and relatively short period of administration for treatment. Based on NK’s unique, comparatively strong fibrinolytic/anticoagulant activity, stability in the gastrointestinal tract, and long bioavailability *in vivo*, NK would appear to offer potential advantages over other currently used agents for treatment and/or prevention of selected diseases processes. Previous study also demonstrated the efficacy of oral NK administration on reduction of systolic/diastolic blood pressure in 73 subjects with pre-hypertension/stage 1 hypertension after 8 weeks of NK intake 2,000 FU daily^19^. Furthermore, velocity of arterial blood flow after acute exercise was enhanced accompanied by NK supplementation in healthy participants^20^. Thus, NK might have an impact on not only fibrinolytic/anticoagulant pathways but also other risk factors for thrombosis, which imply as a NK’s possibility for prevention and/or treatment of the diseases.

In conclusion, a single-dose of NK intake could be a useful fibrinolytic/anticoagulant agent to reduce the risk of thrombosis in humans. Further studies on NK are required to determine the details of metabolism, effective dosage and frequency, and safety for human use. Moreover, human trials demonstrating the clinical benefits of this action are limited, with no outcome data is available currently regarding this agent as an alternative to tPA, aspirin, warfarin, or newer anticoagulants.

## Additional Information

**How to cite this article**: Kurosawa, Y. *et al.* A single-dose of oral nattokinase potentiates thrombolysis and anti-coagulation profiles. *Sci. Rep.* **5**, 11601; doi: 10.1038/srep11601 (2015).

## Figures and Tables

**Table 1:** Blood parameters before and after a 2,000 F.U. of NK administration or placebo in twelve healthy young males

		**Baseline**	**2 h**	**4 h**	**6 h**	**8 h**
aPTT (sec)	P	30.7 ± 0.5	30.3 ± 0.8	30.5 ± 0.7	30.6 ± 0.9	30.4 ± 1.1
	NK	30.5 ± 0.8	31.3 ± 0.9*	31.6 ± 0.9**	31.3 ± 1.0	30.8 ± 0.9
PT-INR	P	1.06 ± 0.02	1.04 ± 0.03	1.04 ± 0.03	1.07 ± 0.03	1.07 ± 0.04
	NK	1.08 ± 0.03	1.06 ± 0.04	1.08 ± 0.04	1.08 ± 0.04	1.05 ± 0.03
Fibrinogen (mg/dL)	P	191.4 ± 6.6	191.1 ± 7.9	191.4 ± 7.7	195.9 ± 7.1	196.1 ± 7.5
	NK	192.7 ± 7.7	191.7 ± 7.8	192.4 ± 7.3	195.4 ± 7.4	189.8 ± 6.5
FDP (μg/mL)	P	1.72 ± 0.10	1.67 ± 0.11	1.55 ± 0.15	1.74 ± 0.10	1.69 ± 0.15
	NK	1.59 ± 0.17	2.12 ± 0.36	1.86 ± 0.16*	1.87 ± 0.16	2.07 ± 0.27
D-dimer (μg/mL)	P	0.15 ± 0.02	0.11 ± 0.02	0.11 ± 0.02	0.11 ± 0.01	0.10 ± 0.02
	NK	0.14 ± 0.04	0.16 ± 0.04	0.15 ± 0.05	0.19 ± 0.07*	0.21 ± 0.09*
AT (mg/dL)	P	27.4 ± 0.8	27.4 ± 0.8	27.5 ± 0.7	28.0 ± 0.6	27.7 ± 0.8
	NK	27.2 ± 0.8	28.4 ± 0.9*	28.4 ± 0.8*	28.5 ± 0.9	28.0 ± 0.9
Plasminogen (mg/dL)	P	12.7 ± 0.4	12.6 ± 0.4	12.6 ± 0.4	12.5 ± 0.4	12.3 ± 0.4
	NK	12.3 ± 0.4	12.2 ± 0.5	12.1 ± 0.5	12.3 ± 0.4	12.1 ± 0.4
Plasminogen Activity (%)	P	87.1 ± 1.9	87.9 ± 2.8	88.2 ± 2.4	88.8 ± 2.3	88.2 ± 2.3
	NK	86.1 ± 2.1	86.3 ± 2.2	85.8 ± 2.5	86.9 ± 2.7	84.5 ± 2.4
PIC (μg/mL)	P	0.43 ± 0.07	0.37 ± 0.06	0.37 ± 0.07	0.34 ± 0.06	0.36 ± 0.06
	NK	0.42 ± 0.06	0.34 ± 0.05	0.37 ± 0.06	0.33 ± 0.07	0.38 ± 0.06
total PAI-1 (ng/mL)	P	14.6 ± 3.2	16.0 ± 2.8	13.0 ± 2.6	12.9 ± 4.2	12.8 ± 3.2
	NK	10.9 ± 1.8	12.6 ± 1.2	12.1 ± 2.1	13.7 ± 2.5	9.1 ± 2.1
FVII activity (%)	P	76.3 ± 4.0	78.9 ± 4.5	76.3 ± 4.7	76.8 ± 4.5	74.2 ± 4.7
	NK	84.1 ± 4.8	81.7 ± 4.5	78.4 ± 4.5	78.6 ± 4.4	74.5 ± 4.3
FVIII activity (%)	P	93.2 ± 4.3	92.0 ± 5.1	91.7 ± 4.9	91.9 ± 5.6	90.7 ± 5.1
	NK	90.6 ± 5.9	87.4 ± 6.2	83.7 ± 5.3*	83.2 ± 5.0*	84.7 ± 4.8
RBC (×10^4^/μL)	P	473.3 ± 9.4	471.1 ± 10.2	470.2 ± 10.5	472.9 ± 11.5	469.5 ± 11.1
	NK	472.6 ± 8.9	470.5 ± 9.8	471.7 ± 9.1	473.0 ± 9.3	467.0 ± 9.6
WBC (μL)	P	4883.3 ± 275.8	4569.0 ± 322.8	5285.1 ± 595.7	5407.2 ± 603.8	5568.1 ± 576.3
	NK	5108.3 ± 355.6	4911.5 ± 315.9	5039.7 ± 355.4	5014.1 ± 372.3	5220.4 ± 405.1
PLT (×10^4^/μL)	P	22.5 ± 1.4	22.9 ± 1.4	23.7 ± 1.6	23.0 ± 1.4	23.8 ± 1.6
	NK	23.4 ± 1.4	23.2 ± 1.2	23.1 ± 1.3	22.9 ± 1.3	23.2 ± 1.3
Protein (g/dL)	P	7.0 ± 0.1	7.0 ± 0.1	7.0 ± 0.1	7.1 ± 0.1	7.1 ± 0.2
	NK	7.0 ± 0.1	7.1 ± 0.1	7.0 ± 0.1	7.0 ± 0.1	7.1 ± 0.1
Glucose (mg/dL)	P	86.8 ± 5.3	74.1 ± 3.4	75.1 ± 3.8	103.6 ± 7.8	65.1 ± 3.0
	NK	92.6 ± 6.1	74.4 ± 3.1	79.5 ± 2.6	95.0 ± 8.6	66.1 ± 2.5

Values are mean ± SEM. **p < 0.01, *p < 0.05 versus placebo.
